# Predicting the Flory-Huggins χ Parameter for Polymers with Stiffness Mismatch from Molecular Dynamics Simulations

**DOI:** 10.3390/polym8060241

**Published:** 2016-06-22

**Authors:** Daniel J. Kozuch, Wenlin Zhang, Scott T. Milner

**Affiliations:** Department of Chemical Engineering, The Pennsylvania State University, University Park, PA 16802, USA; djk5294@psu.edu (D.J.K.); wwz5031@psu.edu (W.Z.)

**Keywords:** polymers, Flory–Huggins theory, bead-spring chain, molecular dynamics, coarse grain, chain stiffness

## Abstract

The Flory–Huggins *χ* parameter describes the excess free energy of mixing and governs phase behavior for polymer blends and block copolymers. For chemically-distinct nonpolar polymers, the value of *χ* is dominated by the mismatch in cohesive energy densities of the monomers. For blends of chemically-similar polymers, the entropic portion of *χ*, arising from non-ideal local packing, becomes more significant. Using polymer field theory, Fredrickson et al. predicted that a difference in backbone stiffness can result in a positive *χ* for chains consisting of chemically-identical monomers. To quantitatively investigate this phenomenon, we perform molecular dynamic (MD) simulations for bead-spring chains, which differ only in stiffness. From the simulations, we apply a novel thermodynamic integration to extract *χ* as low as $10^{-4}$ per monomer for blends with stiffness mismatch. To compare with experiments, we introduce a standardized effective monomer to map real polymers onto our bead-spring chains. The predicted *χ* agrees well with experimental values for a wide variety of pairs of chemically-similar polymers.

## 1. Introduction

Polymer mixtures have been an immensely useful set of materials for many years and continue to provide unique properties in many modern applications [1,2]. Block copolymer systems in particular have been given special attention in recent years, as their nanoscale self-assembly properties are of great interest for microelectronics and photovoltaics [3,4,5]. Since the performance and uses of these materials depend strongly on the the final morphology, a better understanding of the physical laws governing polymer mixing is critical to optimizing next-generation materials [6]. Of fundamental interest is the phase behavior of polymer blends, governed by the degree of polymerization (*N*), composition (ϕ), molecular architecture and monomer interactions [7,8]. Enthalpic and packing effects arising from monomer interactions and molecular architecture give rise to non-ideal mixing effects, which can be troublesome to measure and challenging to model.

### 1.1. Flory–Huggins χ Parameter

The Flory–Huggins *χ* parameter was introduced to summarize these non-ideal contributions to the free energy [9]. The most common method to experimentally determine the *χ* parameter uses random phase approximation (RPA) theory to relate *χ* to the composition fluctuations of a polymer blend melt [10]. These composition fluctuations can be measured using small angle neutron scattering (SANS) and *χ* can be extracted from the scattering data [11]. For strongly-incompatible mixtures, *χ* can be determined through X-ray or neutron reflectivity from flat interfaces between phase-separated polymers [12].

Experimental results for *χ* are typically reported as a function of temperature *T* in the form:

(1) $$\chi \left(T\right)=\chi_{S}+\chi_{H}/T$$

in which the temperature-independent contribution $\chi_{S}$ is often assumed to be of entropic origin, while the term $\chi_{H}/T$ is presumed to be of enthalpic origin.

For chemically dissimilar polymers, *χ* is often dominated by the enthalpic term; for nonpolar polymers, this term can be reasonably understood as a consequence of the mismatch in cohesive energy density between different monomers. A large mismatch in cohesive energy density results in a larger *χ* value and, hence, a greater driving force for demixing [13,14]. However, while chemically-similar polymer systems with small cohesive energy mismatch (i.e., saturated hydrocarbons) are more likely to be miscible, *χ* does not vanish, and demixing can still occur for sufficiently long chains. The entropic term $\chi_{S}$ must therefore be considered to understand and predict demixing of chemically-similar polymers.

### 1.2. Entropic Contributions to χ

Entropic contributions to the *χ* parameter arise from non-ideal packing in the melt. Architectural and geometric differences between polymers (such as chain stiffness, diameter and monomer shape) prevent chains in a mixture from occupying the same configurations they explore in a pure phase. As a result, there can be an entropic penalty for mixing and, hence, a driving force for phase separation. Using polymer field theory, Fredrickson, Liu and Bates elegantly confirmed this idea, predicting that a mismatch in chain stiffness for otherwise identical polymer chains can result in a significant positive *χ*. They were able to predict a functional form for the dependence of *χ* on stiffness difference, although with a prefactor that depended strongly on the short-distance cutoff at the statistical segment length scale, and hence, on microscopic details beyond the domain of a continuum field theory [15].

In the present work, our goal is to obtain *χ* for chains of different stiffness as considered by Fredrickson et al., but with a complementary approach using coarse-grained molecular dynamics (MD) simulations. To represent such a blend in the simplest way possible, we consider chains with identical non-bonded interactions, for which the only difference is the backbone stiffness of the two species. For such a system, we expect on physical grounds that enthalpic contributions to *χ* arising from differences in non-bonded interactions between monomers will be negligible, and contributions *χ* should be dominated by packing effects on the entropy.

### 1.3. Challenges of Calculating χ from Simulation

Calculating *χ* from simulation is not a simple task. Indeed, the smaller *χ* is, the harder the job. We want to obtain from simulations realistically small values of *χ*, which for entropically-dominated systems of chemically-similar chains can be as small as $10^{-4}$ per monomer. Whatever approach we take, extremely accurate simulation measurements will be required to produce meaningful results. Furthermore, although *χ* is directly related to the excess free energy of mixing, free energies cannot be directly measured in MD simulations, but must be obtained from measurable average quantities by some sort of integration. Here, we briefly discuss existing simulation methods that could in principle be used to obtain *χ* and why they do not meet our needs.

If *χ* is large enough that chains are strongly demixed, MD simulations of the interface between demixed chains can be used to obtain *χ*. The interfacial thickness is governed by the Helfand–Tagami relation [16,17], so that *χ* can be inferred from a good measurement of the equilibrium interfacial thickness. This approach has been developed by Groot and Warren [18]. In more recent work, Chremos et al. show that *χ* as a function of the monomer-monomer interaction strength can be obtained from monitoring the composition of the interface at equilibrium [19]. However, this technique only works for *χ* large enough that the interface between demixed phases is sufficiently sharp that it fits well within the simulation volume and equilibrates sufficiently rapidly that it forms well within the simulation time.

If *χ* is small enough that we cannot simulate chains that are long enough to phase separate, we may consider calculating *χ* by monitoring composition fluctuations in a miscible phase and applying RPA theory. However, to get a large enough amplitude of concentration fluctuations to reliably measure, this operation should be performed not too far from the critical point, which implies that we must have χN not terribly small (here, *N* is the number of monomers). Therefore, to calculate *χ* on the order of $10^{-4}$, we need chains of at least 1000 monomers. The entanglement length $N_{e}$ for bead-spring chains is about $N_{e}=65$ [20], and so, such chains would be nearly 20 $N_{e}$ long, with very long reptation times, completely beyond present simulation capabilities to equilibrate.

In principle, *χ* could be obtained by measuring the ratio of insertion probabilities of a single chain into an equilibrated pure or mixed system, using Widom insertion or related techniques [21]. However, this approach is challenging because as the chain length grows, insertion is greatly inhibited by overlap with other chains present in the melt. Hence, the insertion probability for chain segments of any reasonable length into a melt is very low, and *χ* would be governed by the ratio of two ill-measured small numbers. Several adjustments have been attempted to circumvent this issue, but the difficulty in sampling a reasonable number of interactions leads to relatively high error [22].

A different way to obtain *χ* by inserting chains into a pure or mixed phase is to measure the insertion free energy. In this approach, a single chain is added to a system with a variable “visibility” parameter *λ* that controls the strength of interactions between the added chain and the system. A series of simulations are performed for different *λ* and an integration performed with respect to *λ* to obtain the thermodynamic work to “turn on” the interactions and hence insert the chain. This approach works well for inserting small molecules into fluids [23,24], but would be challenging for determining *χ*, because of the extreme accuracy requirements on the difference of insertion free energies into two very similar systems (the pure and mixed melts).

Lastly, conventional thermodynamic integration is perhaps the simplest method for obtaining free energy and, therefore, *χ*, from simulation. In conventional thermodynamic integration, a series of simulations is performed for a system at different temperatures; the free energy is then computed by integrating the average energy with respect to *β* (inverse temperature) from a convenient reference state, typically a weakly-interacting ideal gas at high temperatures. In essence, to get the free energy of a system, we measure the heat we must add to boil it. For polymer melts, this means our simulations must explore “polymer gases” for which the potentials were surely never designed or tested, which may give us pause. Worse, we obtain *χ* from the very small free energy difference between pure and mixed systems, each of which has a very large free energy with respect to the ideal gas state. Effectively, this approach tries to obtain *χ* by comparing how much heat it takes to boil a pure polymer and a mixture. It is not practical to measure each of these free energies with sufficient accuracy that we can obtain *χ* at a level of $10^{-4}$ per monomer.

### 1.4. Novel Method for Determining χ from Simulation

Because of the deficiencies in existing methods for obtaining *χ* from simulations, we introduce a novel thermodynamic integration method. Instead of varying the temperature, we vary the stiffness of half of the chains in the mixture, and instead of the system energy, the quantity to be measured and integrated is the corresponding thermodynamic derivative. The reference state for the integration is then a mixture in which the two species have the same stiffness.

Using a bead-spring system, we let stiffness be a function of a harmonic bond deflection angle potential, $E_{\theta }$. We are then able to calculate excess free energies and, therefore, *χ*, by simply monitoring the resulting average bond deflection angle, *θ*, of chains in corresponding mixed and pure systems. As we shall see below in Section 2.1, our method is related both to insertion free energy calculations and to conventional thermodynamic integration.

Our method has several key advantages. First, since we are measuring average bond deflection angles, useful information is collected from all chains in the system, rather than just for a single chain, as for insertion free energy methods. This allows us to average over a large number of samples and, ultimately, to calculate reliably very small *χ* values. Additionally, the bond deflection angles equilibrate very quickly, so that we can make many independent measurements in a given simulation time.

Second, our method avoids the use of non-physical reference states. Our reference state is simply a system in which the stiffnesses of the two chain types are the same, resulting in an effectively pure system for which the excess free energy vanishes. A related advantage is that we do not need to evaluate a small difference between large free energies; unlike conventional thermodynamic integration, to measure the excess free energy, we do not need to compare the heat required to boil pure versus mixed chains.

Third, by analyzing a simple bead-spring system, our results can be unambiguously checked against field theory predictions and, yet, can also be compared to experimental *χ* values for chemically-similar polymer pairs. In more chemically-detailed atomistic simulations, as in all real systems, there are a number of confounding factors that must be controlled for when analyzing results. Here, we construct an idealized system for which our results depend *only* on the stiffness of the chains. Because of this simple dependence, the bead-spring system can be mapped to all real polymers using only available physical data.

As a consequence of these advantages, we find that *χ* values as low as $10^{-4}$ per monomer can be reliably obtained as a function of chain stiffness mismatch. In Section 3, we compare our *χ* values to those predicted by field theory and find very good agreement. Mapping our simple system to real systems of chemically-similar polymers, our predictions agree well with experimental results in most cases and are always well within an order of magnitude.

## 2. Method

### 2.1. Theory for Determining χ

The Flory–Huggins equation accounts for non-ideal mixing by introducing the *χ* parameter. For two polymers, A and B, composed of $N_{A}$ and $N_{B}$ segments, the free energy of mixing ΔF is given by:

(2) $$\frac{\beta \Delta F}{N}=\frac{\phi_{A}}{N_{A}}\ln\phi_{A}+\frac{\phi_{B}}{N_{B}}\ln\phi_{B}+\chi \phi_{A}\phi_{B}$$

in which *N* is the total number of segments, $\beta =1/k_{B}T$, and $\phi_{A}$ and $\phi_{B}$ are the volume fractions of Polymers A and B, respectively [25].

Considering only the non-ideal mixing terms, the excess free energy of mixing $\Delta F_{E}$ is simply:

(3) $$\frac{\beta \Delta F_{E}}{N}=\chi \phi_{A}\phi_{B}$$

in which *χ* is defined per chain segment. Physically, *χ* measures the net interaction between A and B segments in intimate contact. If we can obtain $\Delta F_{E}$ from simulations with sufficient accuracy, we can determine *χ*. In the experiment, *χ* often exhibits composition dependence [26]. We will avoid this complication in the present work, by investigating symmetric blend compositions, with $\phi_{A}=\phi_{B}=0.5$.

**Figure 1:**
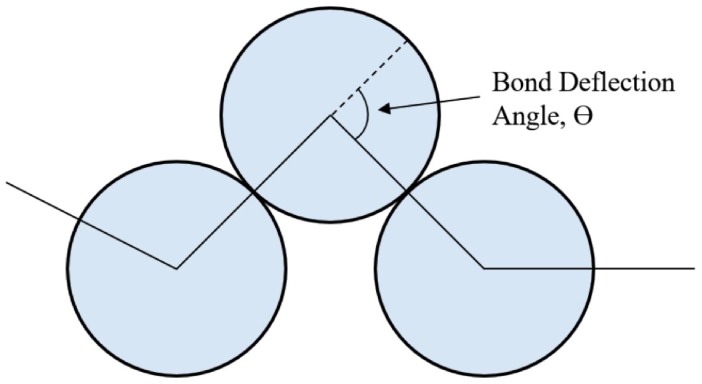
Bead-spring model with bonds (lines connecting the centers of bonded beads). Bond deflection angle *θ* is limited by repulsive interactions between next-neighbor beads to about 120°.

Since we seek *χ* as a function of chain stiffness, we consider a purely repulsive bead-spring system where the backbone stiffness is simulated via a harmonic bond deflection energy E(θ) with angular spring constant *κ*, shown in Equation (4) (see Figure 1). For this system, all non-ideal behavior in the mixture arises from differences in bond angle stiffness.

(4) $$E\left(\theta \right)=\frac{1}{2}\kappa \theta^{2}$$

To determine $\Delta F_{E}$ for the bead-spring system, a relationship between *F* and a measurable quantity must be established. To establish such a relationship, we begin with the partition function *Z*, given by:

(5) $$Z=\sum_{j}e^{-\beta E_{j}}=e^{-\beta F}$$

in which $E_{j}$ is the total energy of some configuration *j*. For example, conventional thermodynamic integration proceeds by taking a derivative with respect to *β* of lnZ=-βF, to obtain the well-known relation:

(6) $$\frac{\partial (\beta F)}{\partial \beta }=\langle E\rangle$$

Here, 〈E〉 denotes the ensemble average of the energy $E_{j}$ in the ensemble described by the partition function *Z*, which can be evaluated as a time-average of a well-equilibrated simulation.

Thus, Equation (6) relates the derivative of the free energy we seek to a measurable equilibrium average. To use this relation to compute *F*, we must evaluate 〈E〉 as a function of *β*, and integrate with respect to *β*. This approach is widely useful, but impractical for computing polymer mixing free energies as discussed above, because in the end, we must subtract two enormous free energies (the work to boil a polymer blend and melt) to obtain a tiny difference.

To avoid this difficulty, we seek some other parameter that we can vary to smoothly connect a polymer blend to some more convenient reference system. We choose the chain stiffness itself, represented by the product βκ (this parameter controls chain stiffness, independent of temperature, since the Boltzmann factor for a bond angle is $e^{-\left(1/2\right)\beta \kappa \theta^{2}}$). We imagine starting with a melt of identical A and B chains, then smoothly increasing the stiffness of the B chains, to reach some desired final value. If we can compute the thermodynamic work required to stiffen the B chains, we can compute the free energy difference between the blend and a reference state of identical chains. Along this path, all of the states are polymer melts, and the free energy changes are therefore small.

This method can also be thought of as related to insertion free energy calculations, in which a “visibility parameter” is smoothly adjusted, to turn on the interactions between the system and a newly-inserted molecule. The work done in turning on the interactions is the insertion free energy. The two key differences between the insertion free energy method and the present approach are: (1) the reference state is not invisible molecules, but identical molecules; and (2) instead of transforming just one molecule from A to B (which would be useful for obtaining the exchange chemical potential), we transform *half* the molecules in the (effectively pure A) reference state from A to B.

To derive a relation analogous to Equation (6), we differentiate instead with respect to βκ at fixed *β*. The only part of the configuration energy $\beta E_{j}$ that depends explicitly on βκ is the bending energy. This allows us to replace $\beta E_{j}$ in the last equality of Equation (7) with the sum over bonds of the harmonic bending energies in the first equality of Equation (8), to obtain:

(7) $$\begin{array}{cc} \frac{\partial \beta F}{\partial \beta \kappa } & =-\frac{1}{Z}\frac{\partial }{\partial \beta \kappa }\sum_{j}e^{-\beta E_{j}}=\frac{1}{Z}\sum_{j}e^{-\beta E_{j}}\frac{\partial (\beta E_{j})}{\partial \beta \kappa } \end{array}$$

(8) $$\begin{array}{cc} & =\frac{1}{Z}\sum_{j}e^{-\beta E_{j}}\frac{\partial }{\partial \beta \kappa }\left(\sum_{i}^{N}\frac{1}{2}\beta \kappa \theta_{i}^{2}\right)=\frac{1}{2}\langle \sum_{i}^{N}\theta_{i}^{2}\rangle \equiv \frac{1}{2}N\langle \bar{\theta^{2}}\rangle \end{array}$$

in which $\langle \bar{\theta^{2}}\rangle$ denotes the arithmetic average of $\langle \theta^{2}\rangle$ over *N* bond angles.

Like conventional thermodynamic integration, Equation (7) relates a derivative of the free energy (with respect to βκ) to an equilibrium average (of the mean-square bond angles). If we evaluate $\langle \bar{\theta^{2}}\rangle$ for a range of βκ values, we can integrate to obtain the difference in free energy between melts of different stiffness.

To compute the excess free energy $\Delta F_{E}$, we generalize this relation to the case of two different bending stiffnesses, $\beta \kappa_{A}$ and $\beta \kappa_{B}$, for the A and B chains. The excess free energy we seek is the difference in free energy between the mixed state and the pure states of the two blend components separately:

(9) $$\Delta F_{E}\left(\kappa_{A},\kappa_{B}\right)=F_{AB}\left(\kappa_{A},\kappa_{B}\right)-\frac{1}{2}\left(F_{A}\left(\kappa_{A}\right)+F_{B}\left(\kappa_{B}\right)\right)$$

Here, $F_{AB}$ is the free energy of the mixture; $F_{A}$ is the free energy of pure chain A; $F_{B}$ is the free energy of pure Chain B; and we assume a 50:50 mixture of otherwise identical chains.

Differentiating Equation (9) with respect to $\beta \kappa_{B}$, and using the relationship of Equations (7) and (8), we obtain:

(10) $$\begin{array}{cc} \frac{1}{N}\frac{\partial \beta \Delta F_{E}}{\partial \beta \kappa_{B}} & =\frac{1}{N}\left(\frac{\partial \beta F_{AB}}{\partial \beta \kappa_{B}}-\frac{1}{2}\frac{\partial \beta F_{B}}{\partial \beta \kappa_{B}}\right) \end{array}$$

(11) $$\begin{array}{cc} & =\frac{1}{2}{\langle {\bar{\theta^{2}}}_{B}\rangle }_{AB}-\frac{1}{2}{\langle {\bar{\theta^{2}}}_{B}\rangle }_{B} \end{array}$$

Here, ${\langle {\bar{\theta^{2}}}_{B}\rangle }_{AB}$ is the ensemble average of the mean-square bond angle for B chains in the AB blend, and ${\langle {\bar{\theta^{2}}}_{B}\rangle }_{B}$ is the ensemble average of the mean-square bond angle for B chains in a pure B melt.

To obtain the excess free energy, we integrate Equation (11) with respect to $\beta \kappa_{B}$, from a reference stiffness $\beta \kappa_{1}$ (the stiffness of the A chains) to the final stiffness $\beta \kappa_{2}$ (the actual stiffness of the B chains):

(12) $$\frac{\beta \Delta F_{E}}{N}=\frac{1}{2}\int_{\beta \kappa_{1}}^{\beta \kappa_{2}}\left({\langle {\bar{\theta^{2}}}_{B}\rangle }_{AB}-{\langle {\bar{\theta^{2}}}_{B}\rangle }_{B}\right)\;d\beta \kappa_{B}$$

From the results for $\beta \Delta F_{E}$, we can compute *χ* using Equation (3).

Note that although we have presented this derivation for the particular case of a 50:50 mixture, it is clear that we can just as easily transform some other fraction ϕ of the total number chains from A to B. The only change in Equation (12) would be to replace the factor of 1/2 by ϕ and to interpret the AB ensemble as one with a fraction ϕ of B chains. In this way, we can obtain the excess free energy and, hence, *χ* for different mole fractions.

In order to evaluate this expression, $\langle \bar{\theta^{2}}\rangle$ needs to be collected for a number of pure and mixed systems with various $\kappa_{A}$, $\kappa_{B}$ pairings. For this study, $\langle \bar{\theta^{2}}\rangle$ is obtained from molecular dynamics simulation, performed using GROMACS software [27,28]. Details are provided in the following section.

### 2.2. Simulation Details

To simulate purely-repulsive beads, the Weeks-Chandler-Anderson (WCA) potential with repulsive interaction energy $E_{LJ}\left(r\right)$ was employed, of the form:

(13) $$E_{LJ}\left(r\right)=\left\{\begin{array}{cc} 4\epsilon \left[{\left(\frac{\sigma }{r}\right)}^{12}-{\left(\frac{\sigma }{r}\right)}^{6}\right]+\epsilon & \;r<2^{1/6}\sigma \\ 0 & \;r\geq 2^{1/6}\sigma \end{array}\right.$$

in which *r* is the distance between the centers of interacting beads, with interaction radius equal to $2^{1/6}\sigma$ [29].

Although our bead-spring model does not represent any particular polymer, to preserve some relationship to atomistic length scales, *σ* was chosen to be 0.2 nm, *ϵ* was set to 2.49 $\times \;10^{3}$ J/mol (1 kT at 300 K) and the bead mass, $M_{b}$, was taken to be 12 g/mol (the mass of a carbon atom). The Lennard–Jones time of the system is therefore $\tau_{LJ}=\sigma {\left(M_{b}/\epsilon \right)}^{1/2}=0.44$ ps [30].

To produce the desired rigid bonds with length equal to the diameter of the beads, a harmonic spring potential was used, of the form:

(14) $$E_{b}\left(r\right)=\frac{1}{2}\kappa_{b}{\left(\frac{r}{2^{1/6}\sigma }-1\right)}^{2}$$

with a high spring constant of $\kappa_{b}=5040$ kT. No dihedral potentials were applied, and bond angles were controlled by a harmonic spring (Equation (4)) with a variable spring constant *κ* on the order of 1 kT. The behavior of the system thus depends on the values of $\kappa_{A}$ and $\kappa_{B}$ chosen for the A and B chains.

**Figure 2:**
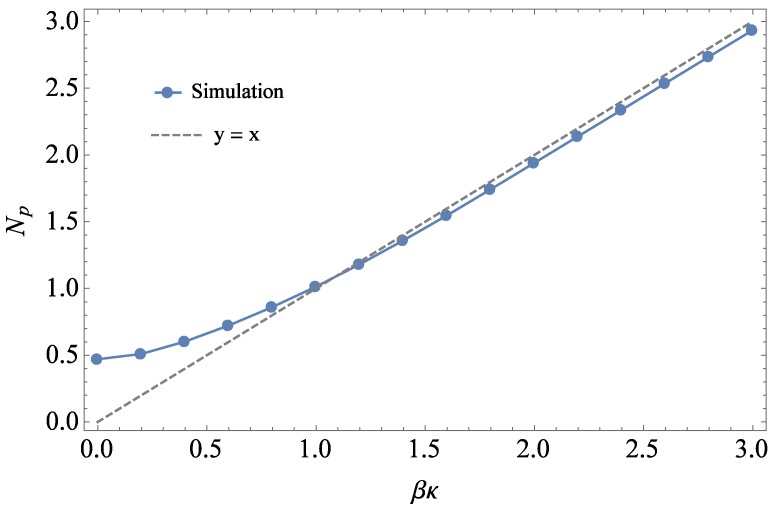
$N_{p}$ (in beads) determined from the simulation for pure systems with βκ ranging from 0 to 3. Results show that for βκ>1, $N_{p}\approx \beta \kappa$.

The value of βκ controls the chain persistence length $N_{p}$, defined as the decay length in monomers for the tangent-tangent correlation function $\langle t_{0}\cdot t_{n}\rangle$, which measures how nearly aligned the tangent $t_{0}$ (at Monomer 0 along the chain) is to the tangent $t_{n}$ (*n* monomers farther along). We use MD simulations to determine the relationship between βκ and the persistence length $N_{p}$ for bead-spring melts. Simulations with βκ varying from 0 to 3 kT were equilibrated, and $N_{p}$ was extracted as the number of bonds for the $\langle t_{0}\cdot t_{n}\rangle$ to decay to 1/e. Figure 2 shows the results, where for βκ>1, $N_{p}\approx \beta \kappa$. Mildly stiff chains with a maximum βκ of three were selected for this work, as stiffer spring constants (βκ>5) caused unwanted nematic ordering. Chain length was consequently set to 40 beads (more than 10-times the maximum persistence length) to ensure that the chains can be considered Gaussian random walks of Kuhn segments. Otherwise, we take our chains as short as possible, to minimize conformational relaxation times and, hence, the equilibration time of the system.

Since the WCA interaction gives no attractive forces between beads, we chose to perform our simulations under NVT conditions, which maintain a constant melt density (in real polymer blends with low *χ* values, we expect negligible volume changes on mixing, so that constant volume simulations are not an unrealistic simplification). To simulate the melt, a bead density of 0.7 beads/$\sigma^{3}$ was used, giving a total system volume of 1543 nm${}^{3}$ [31]. The temperature was fixed at 300 K, and a standard time step of 1 fs was used for all simulations.

Our ability to compute *χ* from simulations depends on obtaining sufficiently accurate values for $\langle \bar{\theta^{2}}\rangle$ (error estimates are discussed below in Section 2.3). As with any simulation, a larger system size and longer duration gives more accurate results. After a significant amount of trial and error, we selected a simulation size of 3375 chains (135,000 beads) and a duration of 200 ns.

**Figure 3:**
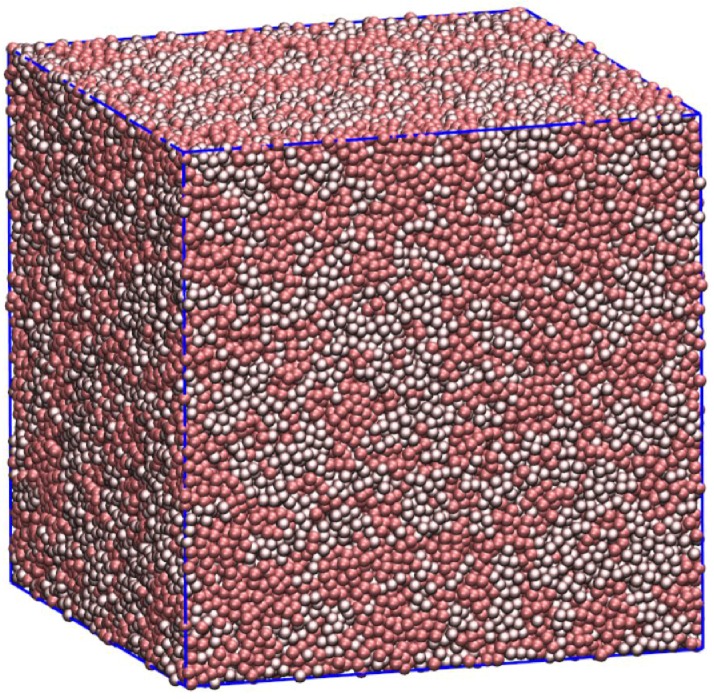
Snapshot of a well-equilibrated blend (flexible chains pink, stiff chains gray). The chains remain well mixed and isotropic throughout the simulation.

To generate a well-equilibrated and disordered initial configuration, a simulation of purely flexible chains (κ=0) was run for over 100 ns. At the beginning of each test, chains were randomly selected from this initial configuration as A and B to form a 50:50 mixture. After the appropriate *κ* values were imposed, all simulations were run for at least 200 ns. Time series for mean-square bond angles relaxed within 10 ns or so to equilibrium. Figure 3 depicts a well-equilibrated system of flexible (lower βκ) and stiff (higher βκ) chains. We verify that the system is equilibrated with standard tests for polymer simulations (chains can diffuse a distance of order their own size, and chain radii of gyration fluctuate repeatedly about their average values).

### 2.3. Analysis of Simulation Data

**Figure 4:**
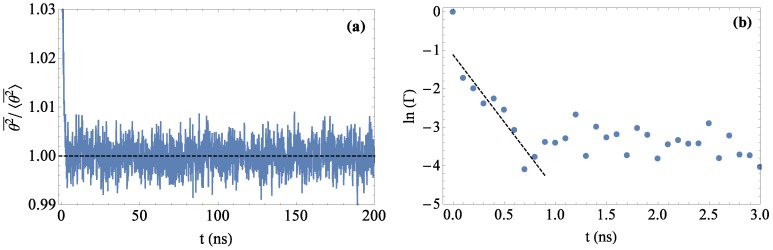
(**a**) The mean-square bond deflection angle, $\bar{\theta^{2}}$, quickly reaches equilibrium. The dashed black line is the ensemble average, $\langle \bar{\theta^{2}}\rangle =0.73$ radians. (**b**) The correlation function for a selected simulation exponentially decays to near zero within 1 ns of simulation time.

From the simulation, the average bond angles $\bar{\theta^{2}}$ for both types of bonds were extracted as a function of time (example results shown in Figure 4a). Non-equilibrium values from 0 to 10 ns were discarded, and $\langle \bar{\theta^{2}}\rangle$ was determined by averaging over the remaining well-equilibrated data.

To estimate the error in $\langle \bar{\theta^{2}}\rangle$, we employ the autocorrelation function, Γ(t) in Equation (15), to determine the number of statistically-independent measurements [21]. The correlation function for $\langle \bar{\theta^{2}}\rangle$ is defined as:

(15) $$\Gamma \left(t\right)=\frac{\sum_{i=1}^{n}\left({\bar{\theta^{2}}}_{i}-\langle \bar{\theta^{2}}\rangle \right)\left({\bar{\theta^{2}}}_{i+t}-\langle \bar{\theta^{2}}\rangle \right)}{\sum_{i=1}^{n}{\left({\bar{\theta^{2}}}_{i}-\langle \bar{\theta^{2}}\rangle \right)}^{2}}$$

in which *n* is the total number of measurements taken at different times, *t*, during the simulation.

We calculate the autocorrelation time *τ* as the negative inverse slope of lnΓ(t), as the decay of Γ(t) is reasonably described as a decaying exponential $e^{-t/\tau }$ (Figure 4b). Taking *τ* to be the time required for a single independent measurement, we have effectively T/τ independent measurements. Then, the error *δ* associated with our value of $\langle \bar{\theta^{2}}\rangle$ can be calculated in the usual way as:

(16) $$\delta ={\left(\frac{\nu }{T/\tau }\right)}^{1/2}$$

where *ν* is the variance of the time series and *T* is the total sampling time. With an average *τ* of approximately 0.3 ns and a simulation time of at least 200 ns, each $\langle \bar{\theta^{2}}\rangle$ result represents an average of over 600 independent measurements. In addition, the maximum autocorrelation time for the end-to-end distance *R* was calculated to be smaller than 2 ns, indicating that the system was well equilibrated.

**Figure 5:**
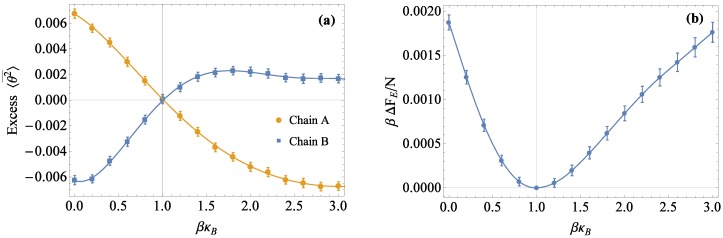
(**a**) Excess $\langle \bar{\theta^{2}}\rangle$ in radians as a function of βκ for simulations with a reference chain of stiffness $\beta \kappa_{A}=1$. The error shown was calculated from the autocorrelation time. (**b**) $\beta \Delta F_{E}/N$ determined via integration of excess $\langle \bar{\theta^{2}}\rangle$. $\Delta F_{E}$ is zero by definition where the reference and variable chain stiffnesses are equal at βκ=1.

Returning to Equation (12), we must compute ${\langle {\bar{\theta^{2}}}_{B}\rangle }_{AB}-{\langle {\bar{\theta^{2}}}_{B}\rangle }_{B}$, which will be referred to as the excess $\langle {\bar{\theta^{2}}}_{B}\rangle$, as a function of $\beta \kappa_{B}$. Example results for excess $\langle {\bar{\theta^{2}}}_{B}\rangle$ with a reference chain stiffness of $\beta \kappa_{A}=1$ are shown in Figure 5a, with error bars computed using Equation (16) (for completeness, Figure 5a also includes excess ${\langle \bar{\theta^{2}}\rangle }_{A}$, although these data are not required for our free energy calculations). Note that the excess ${\langle \bar{\theta^{2}}\rangle }_{B}$ is negative when B is stiffer and positive when B is more flexible. From Equation (12), this means $\Delta F_{E}$, and hence, *χ* will always be positive, whether we take $\beta \kappa_{2}>\beta \kappa_{1}$ (B stiffer than A) or vice versa.

Integrating the excess ${\langle \bar{\theta^{2}}\rangle }_{B}$ using Equation (12), we obtain the excess free energy $\beta \Delta F_{E}/N$ as a function of $\beta \kappa_{B}$, as shown in Figure 5b. As expected, $\beta \Delta F_{E}/N=0$ where $\beta \kappa_{B}=\beta \kappa_{A}$, since this corresponds effectively to a pure system in which all chains have the same stiffness. Since $\beta \Delta F_{E}/N$ is calculated by numerically integrating the excess ${\langle \bar{\theta^{2}}\rangle }_{B}$, the error bars in Figure 5b are calculated from the error bars in Figure 5a, according to rules for additive functions.

## 3. Results

**Figure 6:**
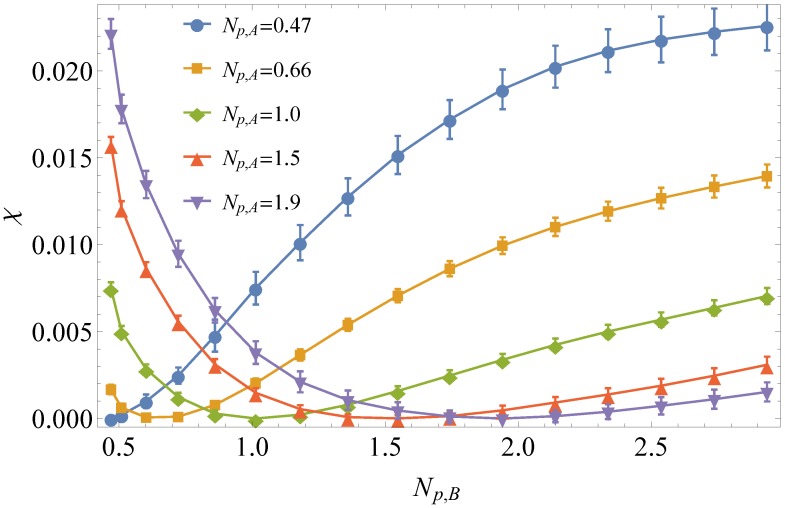
*χ* per bead as a function of the persistence length of the variable chain, $N_{p,B}$.

Using the method outlined above, ${\langle {\bar{\theta^{2}}}_{B}\rangle }_{AB}-{\langle {\bar{\theta^{2}}}_{B}\rangle }_{B}$ was obtained for five sets of simulations with $\beta \kappa_{A}=$ 0, 0.5, 1.0, 1.5 and 2. For each simulation set, $\beta \kappa_{B}$ was varied from zero to three in steps of 0.2. Since $\phi_{A}$ and $\phi_{B}$ were always maintained at 0.5 for the mixed systems, Equations (3) and (12) can be used to determine *χ* as a function of βκ. Converting βκ to $N_{p}$ using the relationship shown in Figure 2, *χ* was then calculated as a function of $N_{p}$, shown in Figure 6.

For our bead-spring system, *χ* is a function of the two independent stiffnesses, $N_{p,1}$ and $N_{p,2}$. To construct a phenomenological fitting function, we identify several requirements that $\chi (N_{p,1},N_{p,2})$ must satisfy. First, *χ* must be zero for $N_{p,1}=N_{p,2}$, since this represents a pure system. Second, $\chi (N_{p,1},N_{p,2})$ must be a symmetric function of its arguments, since the A and B chains are identical other than stiffness, and we assume a symmetric binary blend with $\phi_{1}=\phi_{2}$, so the selection of which polymer is “Polymer 1” and which is “Polymer 2” is arbitrary. Lastly, according to the results shown in Figure 6, *χ* is always positive, indicating an entropic increase in free energy upon mixing.

Based on these requirements, we might hope to expand *χ* in even powers of $N_{p,1}-N_{p,2}$, as:

(17) $$\chi =a_{1}{\left(N_{p,1}-N_{p,2}\right)}^{2}+b_{1}{\left(N_{p,1}-N_{p,2}\right)}^{4}+\ldots$$

which imposes the required symmetry and vanishes when the two chains have the same stiffness. However, the data show that *χ* is also a function of the average stiffness $(N_{p,1}+N_{p,2})/2$, with a tendency towards smaller *χ* values when the average stiffness is larger (at a given stiffness difference). In order to adjust for the decaying value of *χ* as the average stiffness increases, we generalize our fitting form as:

(18) $$\chi =\frac{a_{1}{\left(N_{p,1}-N_{p,2}\right)}^{2}}{{\left(N_{p,1}+N_{p,2}\right)}^{a_{2}}}+\frac{b_{1}{\left(N_{p,1}-N_{p,2}\right)}^{4}}{{\left(N_{p,1}+N_{p,2}\right)}^{b_{2}}}$$

Here, the coefficients $a_{1},a_{2}$ and exponents $b_{1},b_{2}$ are fitting parameters, which we determine by fitting to the simulation results for *χ*.

**Figure 7:**
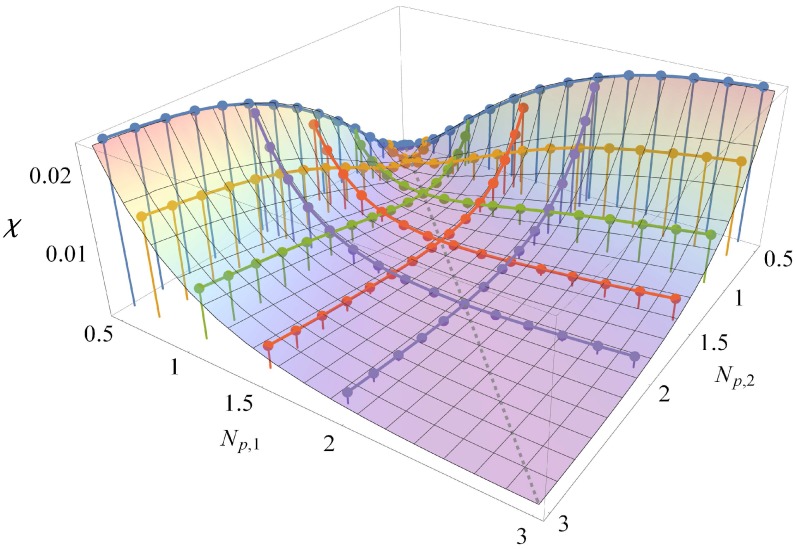
*χ* per bead as a function of the persistence length of both chains. Equation (18) is plotted as a surface over the data points shown in Figure 6. Colors correspond to the colors in Figure 6.

Figure 7 shows the resulting fit, together with the simulation results from Figure 6 (points), plotted as a function of the two stiffnesses $N_{p,1}$ and $N_{p,2}$. Table 1 reports the fitted parameter values. In the figure, each colored curve, which interpolates a set of data points, represents results from an independent set of simulations. Each time two different datasets cross, they do so at a consistent intersection, indicating that our results for *χ* are accurately reproducible. We can use the fitted function $\chi (N_{p,1},N_{p,2})$ to interpolate our *χ* values, allowing us to make predictions for *χ* for bead-spring chains of any stiffnesses within the range spanned by our results.

### 3.1. Comparison to Field Theory

We can compare our results for *χ* between bead-spring chains of different stiffness to analytical predictions from continuum field theory calculations of Fredrickson, Liu and Bates [15]. Fredrickson et al. predicted the functional form that relates the ratio of backbone stiffnesses for a binary polymer blend to $\alpha_{\epsilon }$, which is the same as our entropic *χ*:

(19) $$\alpha_{\epsilon }=\frac{\Lambda^{3}}{24\pi^{2}}{\left[\frac{1-{\left(\beta_{A}/\beta_{B}\right)}^{2}}{\phi +\left(1-\phi \right){\left(\beta_{A}/\beta_{B}\right)}^{2}}\right]}^{2}$$

The prefactor in Equation (19) includes a strongly cutoff-dependent coefficient Λ, with dimensions of inverse length, which is expected to scale on dimensional grounds as $\Lambda ∼{\left(b_{A}b_{B}\right)}^{-1/2}$, where $b_{A}$ and $b_{B}$ are the statistical segment lengths of the A and B chains. This coefficient cannot be reliably calculated within the continuum theory, which does not represent short-distance details of monomer shape and packing. Otherwise, the functional form of Equation (19) is predicted to be universal; and has never been systematically tested by comparison to simulations.

In Equation (19), $\beta_{i}$ is defined by the relation $\beta_{i}^{2}=b_{i}^{2}/6v_{i}$, where $b_{i}$ is the statistical segment length and $v_{i}$ the repeat unit volume of species *i*. For our bead-spring system, $v_{A}$ equals $v_{B}$, and the monomer segment lengths are identical, so that the ratio $\beta_{A}^{2}/\beta_{B}^{2}$ can be replaced by $N_{p,A}/N_{p,B}$. (To see this, recall that the mean-square end-to-end distance $\langle R^{2}\rangle =Nb^{2}$ defines the statistical segment length *b*, while for semiflexible chains, the persistence length $\ell_{p}$ is defined by $\langle R^{2}\rangle =2N\ell_{p}$. Taking the ratio of $R^{2}$ for equal-length A and B chains, we have $\left(b_{A}^{2}/b_{B}^{2}\right)=\ell_{p,A}/\ell_{p,B}$. With identical monomer volumes and segment lengths, this gives $\beta_{A}^{2}/\beta_{B}^{2}=N_{p,A}/N_{p,B}$)

Additionally, note that Λ, with its units of inverse length, serves to dimensionalize $\alpha_{\epsilon }$, which is defined per unit volume in [15]. In the present work, we define *χ* per bead, and so, Λ for comparison to our system is a dimensionless constant. An advantage of using molecular dynamics simulation is that short-range details of the microscopic model are properly accounted for, without any need for imposed cutoffs. In a sense, simulation results could be interpreted as a way to determine the cutoff-dependent prefactor of Equation (19), if the overall functional form for *χ* describes the simulation values.

**Figure 8:**
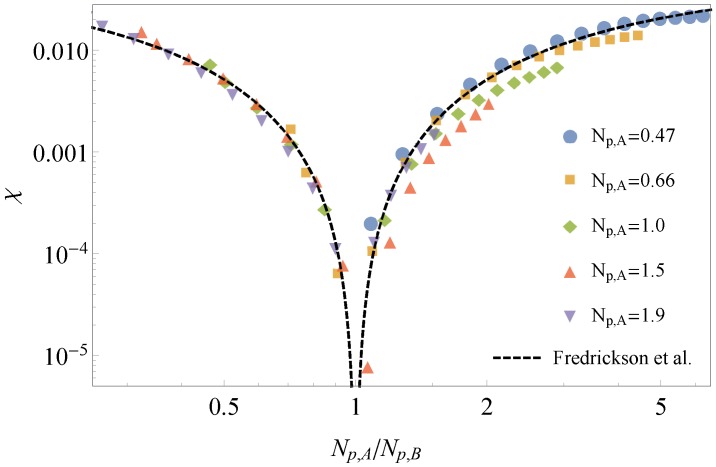
Field theory predictions for *χ* ([15]) agree rather well with simulation results for semiflexible bead-spring chains using our novel thermodynamic integration method.

Figure 8 displays our results together with the field theory prediction, plotted log-log simply to capture the wide range of values of *χ* and stiffness ratio $N_{p,A}/N_{p,B}$. The simulation results and field theory predictions agree rather well, without any adjustment other than the choice of Λ=1.4. The agreement is not perfect; a closer look shows that *χ* does not depend only on the stiffness ratio, but families of points for different values of $N_{p,A}$ do not quite collapse onto a single curve. However, these modest deviations may reflect the conclusion of [15] that the prefactor Λ is non-universal, and may depend on microscopic details other than the stiffness ratio $N_{p,A}/N_{p,B}$ (such as, e.g., the average stiffness).

### 3.2. Comparison to Experiments

*χ* has been experimentally determined for many binary polymer mixtures [32]. By selecting mixtures in which the polymers have a similar chemical makeup with no strong attraction or repulsion (e.g., consisting of saturated hydrocarbons), we are able to compare the results for the bead-spring system to real polymers. However, mapping the bead-spring system to real polymers and relating the calculated entropic *χ* per monomer to the measured *χ* require several major assumptions.

To relate the bead-spring model to real polymers, a correspondence must be made between some portion of a polymer chain and a bead. Many such correspondences can be proposed, between the bead diameter and various microscopic dimensions of the real polymer. We set the bead diameter equal to the average chain diameter, $D_{avg}$, of the real polymers. The simplifications of this mapping are evident, as we ignore the effects of differences in monomer size between the two real chains and of the non-spherical shape of real monomers.

**Figure 9:**
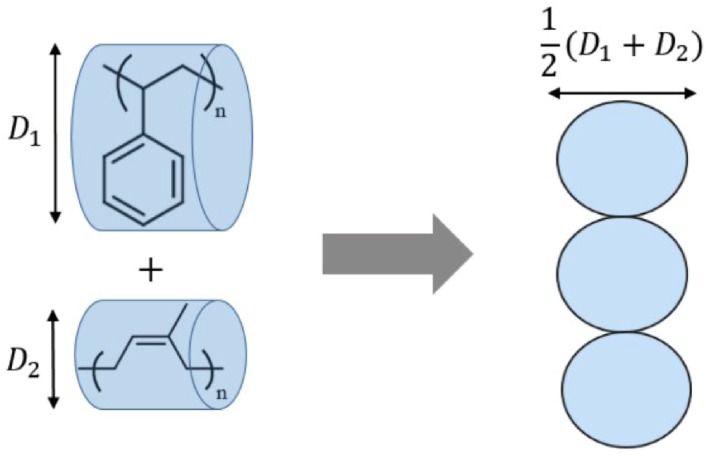
To relate the bead-spring model to real polymers, the bead diameter is taken as the average diameter of the real chains (such as polystyrene and polyisoprene, shown in the figure).

We define the diameter *D* of real polymers by representing the volume *V* a chain displaces in the melt as a long cylinder, with length equal to the fully-extended chain length *L* (see Figure 9). This implies $\pi {\left(D/2\right)}^{2}L=V$. Dividing by the number of monomers and rearranging gives:

(20) $$D=2{\left(\frac{V_{0}}{\pi L_{0}}\right)}^{1/2}$$

in which $V_{0}$ and $L_{0}$ are respectively the displaced volume and fully-extended length per monomer.

The volume per monomer is computed from $V_{0}=M_{0}/\left(\rho N_{A}\right)$, in which $M_{0}$ is the monomer molar mass, *ρ* the density and $N_{A}$ Avogadro’s number. The fully-extended length per monomer was determined using the software package Avogadro [33], which draws reasonably accurate molecular geometries.

A different, but equally appealing correspondence between real polymers and the bead-spring model would choose the bead diameter so that the displaced volumes and fully-extended lengths of the real and bead-spring chains were equal. This amounts to requiring:

(21) $$M/\left(\rho N_{A}\right)=\left(1/0.7\right)\left(4\pi /3\right){\left(D/2\right)}^{3}\left(L/D\right)$$

in which the fully-extended lengths *L* of the real and bead-spring chains are equal. Here, the left side is the displaced volume of a real chain (where *M* is the total molecular weight of the real chain), and the right side is the displaced volume of a bead-spring chain (the factor of 1/0.7 accounts for the fact that the bead volume fraction in the bead-spring melt is 0.7). Fortuitously, this gives the same expression for *D* as Equation (20), times a factor very close to unity ($\sqrt{1.05}$).

The persistence length $\ell_{p}$ of a real polymer is determined by comparing its measured mean-square end-to-end distance $\langle R^{2}\rangle$ to the theoretical result for a worm-like chain. A useful table of chain dimensions is given in [34], presented as values of $\langle R^{2}\rangle /M$, the mean square end-to-end distance per chain mass (in units of nm${}^{2}$ per g/mol).

The result for a worm-like chain is $\langle R^{2}\rangle =L\ell_{K}$, in which $\ell_{K}$ is the Kuhn length ($\ell_{K}=2\ell_{p}$). Combining these results, we have:

(22) $$\ell_{p}=\frac{\langle R^{2}\rangle /M}{2L_{0}/M_{0}}$$

Finally, the persistence length in beads $N_{p}$ is obtained from:

(23) $$N_{p}=\frac{\ell_{p}}{D_{avg}}$$

The standard reference volume for experimental values of *χ* is $V_{r}=0.1$ nm${}^{3}$ [32], whereas our simulation results for *χ* are “per bead”. To connect the two, we equate the corresponding intensive properties, i.e., $\chi /V_{b,avg}$ for the bead-spring model is compared to experimental values of $\chi /V_{r}$. Finally, we note that although we have selected polymer pairs to be as chemically similar as possible, there could still be enthalpic contributions to the experimental *χ*, with no correspondence to our bead-spring system, in which the non-bonded interactions of the A and B chains are identical. Hence, we compare only the experimental “entropic” part $\chi_{S}$ to our simulations. To make this comparison, following the above discussion, we compare our simulation results to:

(24) $$\chi =\chi_{S}\frac{V_{b,avg}}{V_{r}}$$

in which $V_{b,avg}=\left(4/3\right)\pi {\left(D_{avg}/2\right)}^{3}$ is the volume of the bead with diameter $D_{avg}$.

**Figure 10:**
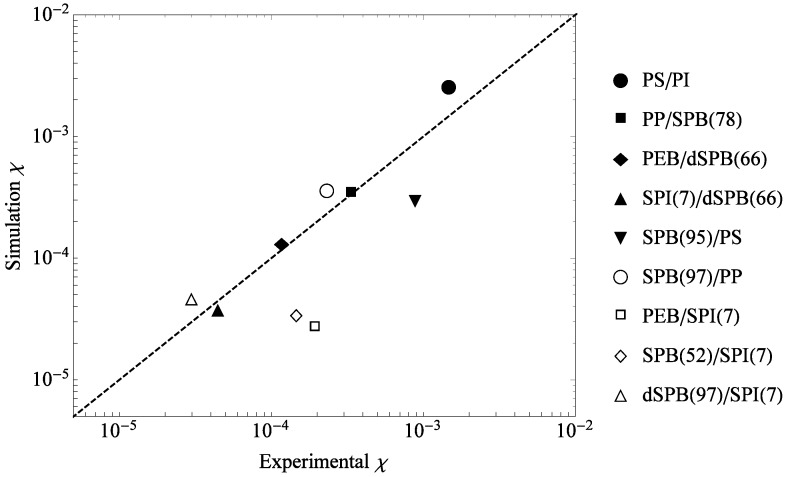
Scatter plot of *χ* per bead predicted by the simulation model vs. the entropic portion of experimental *χ* from the literature. Numbers in parentheses following the polymer labels indicate the degree of branching in mol %. A “d” preceding a polymer label indicates a deuterated polymer. Data can be found in Table 2 and Table 3. Polymer abbreviations are defined in Table 4.

In this way, experimentally-measured $\chi_{S}$ values can be compared to *χ* predicted by the simulation model (Equation (18)). The results are plotted in Figure 10. Given the idealized nature of the bead spring model and the simplicity of the mapping method, the quantitative agreement between the predicted and measured values for many mixtures is remarkable. All results are within an order of magnitude estimate, and six of the nine polymer pairs studied are in close agreement with bead-spring model results.

All data for Figure 10 are given in Table 2 and Table 3. Parentheses indicate the degree of branching (e.g., SPI(75) indicates that 75 mol % of monomers are branched). All parameter values for polymers with significant (greater than 10%) partial branching are calculated from a weighted average of the fully-linear and fully-branched values (e.g., $\ell_{p}$ for SPB(78) = 0.78 $\ell_{p}$ of SPB(100) + 0.22 $\ell_{p}$ of SPB(0)). For a list of polymer structures, please see Table 19.2 of Mark’s Physical Properties of Polymers Handbook , Second Edition [32].

## 4. Conclusions

In this contribution, we report the development and application of a novel thermodynamic integration scheme in which we integrate over chain stiffness instead of temperature. Using molecular dynamics simulations, we demonstrate that precise excess free energies can be determined by analyzing the relationship between measured bond deflection angles and chain stiffness. From the excess free energy, we extract values of the Flory–Huggins *χ* parameter as low as 10${}^{-4}$ per monomer for polymers with stiffness mismatch.

Since we employ a bead-spring system in which the only free parameter is stiffness, our method is particularly well suited for comparison to field theory predictions. Our results are in good agreement with the previously untested field theory prediction of Fredrickson et al. Furthermore, by mapping our bead-spring chains to systems of real polymers and comparing our results with experimentally-determined *χ*, we demonstrate that our predictions are surprisingly accurate and within an order of magnitude in all cases. This consistency with experimental results is impressive given the simplicity of our model and mapping method.

The usefulness of our method arises from the following factors: (1) The method collects information from the entire system of thousands of chains instead of a single selected chain or small interface set. As a result, the data derived from simulation are statistically relevant; (2) It uses a physically-relevant reference state that does not require unrealistic high temperature systems; (3) The simplicity of our bead-spring model and mapping method means that our results are applicable to many polymer systems and only require available physical information, not expensive atomistic simulation.

Our general approach can be described as computing the thermodynamic work required to “morph” one species of polymer into another. This approach is not limited to predicting *χ* as a function of stiffness mismatch. Rather, thermodynamic relations analogous to those developed here can be used to calculate excess free energies via thermodynamic integration for any Hamiltonian that can be morphed between species. Future work will explore the calculation of *χ* as a function of more complex interactions.

## Figures and Tables

**Table 1:** Parameters for fitting $\chi (N_{p,1},N_{p,2})$ to simulation data.

Parameter	$a_{1}$	$a_{2}$	$b_{1}$	$b_{2}$
Value	0.162	5.40	0.0563	2.59

**Table 2:** Data for real polymers.

Polymer	Temperature (K)	*ρ* (g·cm${}^{-3}$)	$M_{0}$ (g·mol${}^{-1}$)	*L* (nm)	$V_{0}$ (nm${}^{3}$)	$\langle R^{2}\rangle /M$ (nm${}^{2}$·mol·g${}^{-1}$)	$\ell_{p}$ (nm)	*D* (nm)	$V_{b}$ (nm${}^{3}$)
PS	413	0.969	104	0.252	0.178	$4.34\times 10^{-3}$	0.896	0.402	$3.37\times 10^{-2}$
SPS	413	0.920	109	0.252	0.197	$3.23\times 10^{-3}$	0.699	0.442	$4.54\times 10^{-2}$
PI	298	0.913	68.1	0.501	0.124	$6.79\times 10^{-3}$	0.462	0.1976	$4.04\times 10^{-3}$
SPI(75)	413	0.810	70.2	0.252	0.144	$5.29\times 10^{-3}$	0.735	0.324	$1.77\times 10^{-2}$
SPI	298	0.856	70.2	0.506	0.136	$9.24\times 10^{-3}$	0.640	0.216	$5.26\times 10^{-3}$
PB(100)	300	0.890	54.1	0.252	0.101	$6.61\times 10^{-3}$	0.709	0.226	$6.10\times 10^{-3}$
PB	298	0.900	54.1	0.501	0.100	$7.58\times 10^{-3}$	0.410	0.159	$2.11\times 10^{-3}$
SPB(98)	298	0.784	56.1	0.252	0.119	$6.40\times 10^{-3}$	0.712	0.266	$9.96\times 10^{-3}$
SPB	413	0.784	56.1	0.506	0.119	$1.25\times 10^{-2}$	0.693	0.1886	$3.52\times 10^{-3}$
PEB	298	0.861	84.2	0.506	0.162	$7.25\times 10^{-3}$	0.603	0.258	$8.92\times 10^{-3}$
PP	298	0.852	84.2	0.506	0.164	$6.64\times 10^{-3}$	0.552	0.262	$9.21\times 10^{-3}$

**Table 3:** Comparing *χ* from experiment and simulation.

Polymer Pair	$\ell_{p,1}$ (nm)	$\ell_{p,2}$ (nm)	$D_{avg}$ (nm)	$N_{p,1}$	$N_{p,2}$	$\chi_{S}$	$V_{b,avg}$ (nm${}^{3}$)	*χ* (Experimental)	*χ* (Simulation)
PS/PI	0.896	0.462	0.299	2.99	1.54	$7.85\times 10^{-3}$	$1.89\times 10^{-2}$	$1.48\times 10^{-3}$	$2.56\times 10^{-3}$
PP/SPB(78)	0.552	0.708	0.255	2.17	2.78	$3.81\times 10^{-3}$	$8.88\times 10^{-3}$	$3.38\times 10^{-4}$	$3.39\times 10^{-4}$
PEB/dSPB(66)	0.603	0.705	0.249	2.42	2.83	$1.41\times 10^{-3}$	$8.35\times 10^{-3}$	$1.18\times 10^{-4}$	$1.31\times 10^{-4}$
SPI(7)/dSPB(66)	0.647	0.705	0.236	2.75	2.99	$6.90\times 10^{-4}$	$6.51\times 10^{-3}$	$4.49\times 10^{-5}$	$3.78\times 10^{-5}$
SPB(95)/PS	0.711	0.896	0.332	2.14	2.70	$4.11\times 10^{-3}$	$2.17\times 10^{-2}$	$8.91\times 10^{-4}$	$2.98\times 10^{-4}$
SPB(97)/PP	0.711	0.552	0.262	2.71	2.10	$2.44\times 10^{-3}$	$9.58\times 10^{-3}$	$2.34\times 10^{-4}$	$3.59\times 10^{-4}$
PEB/SPI(7)	0.603	0.647	0.244	2.47	2.65	$2.75\times 10^{-3}$	$7.09\times 10^{-3}$	$1.95\times 10^{-4}$	$2.68\times 10^{-5}$
SPB(52)/SPI(7)	0.703	0.647	0.230	3.05	2.81	$2.43\times 10^{-3}$	$6.06\times 10^{-3}$	$1.47\times 10^{-4}$	$3.41\times 10^{-5}$
dSPB(97)/SPI(7)	0.711	0.647	0.248	2.87	2.61	$4.01\times 10^{-4}$	$7.51\times 10^{-3}$	$3.01\times 10^{-5}$	$4.67\times 10^{-5}$

**Table 4:** Polymer Abbreviations.

Abbreviation	Polymer
PS	polystyrene
SPS	saturated polystyrene; poly(vinylcyclohexane)
PI	1,4 polyisoprene
SPI	saturated 1,4 polyisoprene; alternating poly ethylene-co-propylene
PB	1,4 polybutadiene
SPB	saturated 1,4 polybutadiene; polyethylene
PEB	poly(ethyl butylene)
PP	polypropylene
